# Sex‐specific analysis of renal cell carcinoma histology and survival in Japan: A population‐based study 2004 to 2016

**DOI:** 10.1002/hsr2.142

**Published:** 2019-12-20

**Authors:** Masayoshi Zaitsu, Satoshi Toyokawa, Takumi Takeuchi, Yasuki Kobayashi, Ichiro Kawachi

**Affiliations:** ^1^ Department of Public Health, Graduate School of Medicine The University of Tokyo Tokyo Japan; ^2^ Department of Social and Behavioral Sciences Harvard T.H. Chan School of Public Health Boston Massachusetts; ^3^ Department of Urology Kanto Rosai Hospital Kawasaki Japan

**Keywords:** kidney cancer, pathology, population‐based, sex difference, survival

## Abstract

**Background and aims:**

In Western countries, sex differences in renal cell carcinoma (RCC) histology and survival have been previously described: compared with men, clear cell subtype is more common but overall prognosis is better among women. The goal of the present study was to examine sex differences in RCC histology and survival in Japan, using a large‐scale population‐based data set.

**Methods:**

With the use of a population‐based cancer registry in Japan (2004‐2016), patients with primary RCC were followed for 5 years (median follow‐up time 2.1 years). We distinguished histological subtypes of clear cell, papillary, and chromophobe from “others” subtype. Sex‐specific prevalence ratio (PR) for each histological subtype was estimated by Poisson regression with robust variance, adjusted for age and year of diagnosis. Sex‐specific survival rates were estimated by Cox proportional hazard regression, adjusted for age, year of diagnosis, histological subtypes, and other prognostic variables, with multiple imputation.

**Results:**

The prevalence of clear cell and “others” subtypes was similar between men and women among all the 5265 study subjects during the 12 years of study (clear cell, male 88.6% vs female 87.1%; “others“, male 5.3% vs female 5.3%). However, papillary subtype was less common among women than men (male 4.6% vs female 2.8%; PR = 0.63; 95% CI, 0.45‐0.88), while chromophobe subtype was more common among women (male 1.6% vs female 4.8%; PR = 3.18; 95% CI, 2.26‐4.47). Although “others” subtype (but not papillary/chromophobe subtypes) independently predicted prognosis (HR = 1.74; 95% CI, 1.32‐2.30), no sex differences were observed in RCC survival.

**Conclusion:**

We did not observe a statistically significant difference in the prevalence of clear cell subtype between men and women in Japan, which differs from the pattern previously described in Western countries. Sex differences in RCC histology may not affect RCC survival in this population.

## 1 INTRODUCTION

Renal cell carcinoma (RCC) ranks as the sixth most common type of cancer in men and 10th among women, worldwide, accounting for 5% and 3% of overall cancer incidence, respectively.^1^ The incidence rate for RCC in Asian countries, including Japan, is roughly one‐third compared with that in Western countries,^1^ and the lower RCC incidence in Japan may be attributable to the lower prevalence of obesity (a prominent risk for RCC^2^) in that country compared with the Western settings. However, the incidence has been rising in recent years in Japan.^1^, ^2^, ^3^ Advanced imaging modalities, together with changes in lifestyle and behavioral risks (such as smoking, obesity, hypertension, and occupational stress), may underlie the increase in incidence.^3^, ^4^, ^5^

Prognostic differences according to histological subtypes are well described. In the United States (USA), studies using the Surveillance, Epidemiology, and End Results (SEER) database suggest that the most common histological subtype is the clear cell subtype (approximately 80%‐90%), followed by the papillary (approximately 4%‐13%) and chromophobe (approximately 2%‐5%) subtypes.^6^, ^7^, ^8^ Compared with the clear cell subtype, patients with papillary and chromophobe subtypes tend to have better prognosis (hazard ratio [HR] approximately 0.67‐0.98), but patients with other rare histological subtypes (eg, collecting duct and sarcomatoid differentiation) tend to have a poorer prognosis (HR approximately 1.81‐2.21).^8^ Previous single‐center studies in the United States are consistent with this pattern.^9^, ^10^

More recently, studies in Western settings have reported sex differences in RCC histology and survival.^11^, ^12^, ^13^, ^14^ The clear cell subtype is likely more prevalent among women compared with men (88% vs 85%).^11^ In addition, among all incident RCC, the papillary subtype tends to be approximately 0.5 to 0.6 times less prevalent, but the chromophobe subtype tends to be approximately 1.6 to 2.3 times more prevalent among women compared with men.^12^, ^13^ Sex differences in RCC histology, particularly in the clear cell subtype, may be partly attributed to lifestyle and behavioral factors such as obesity,^15^ which is generally more prevalent in women than men across most countries,^16^ and smoking.^17^ However, compared with men, RCC prognosis has been reported to be better among women (HR 0.92) even though the clear cell subtype, which has a more unfavorable prognosis, was more prevalent among women.^11^ Other female clinicopathological features (lower pathological grade, earlier stage of detection, and higher prevalence of chromophobe subtype) linked to favorable prognosis may underlie these paradoxical sex differences in RCC histology and survival.^8^, ^11^, ^12^, ^13^

Reports of sex differences in RCC histology and survival remain scarce in Asian settings. For example, in Japan, although the Japanese Urological Association has reported descriptive statistics of RCC histology and 5‐year overall survival with multicenter data (but not population‐based data),^18^ sex differences in RCC histology and survival have not been previously assessed. One study in South Korea, with 1508 RCC patients, examined sex differences and reported lower prevalence of clear cell subtypes among women compared with men (female 72% vs male 84%) but a nonsignificant sex difference in survival, which contradicts the Western pattern.^19^ However, this was a relatively small, single‐center study, and it was not population based.

Accordingly, the goal of the present study was to examine sex differences in RCC histology and survival in Japan, using a large‐scale population‐based data set with over 5000 RCC patients. We sought to examine whether sex differences exist in RCC histology and whether there are overall survival differences by sex and by histological subtypes in Japan. Also, we sought to determine whether a sex difference in survival, if there is one, persists even after controlling for histological subtypes and other potential prognostic factors such as pathological grade, tumor stage, treatment, socioeconomic status (SES), and smoking habits.

## 2 MATERIALS AND METHODS

### 2.1 Data setting and study subjects

A large, population‐based data set (2004‐2016) of Kanagawa Cancer Registry (KCR), a survey of over nine million people in Kanagawa prefecture that covers approximately 7% of the Japanese population, was used for analysis. Details of the study database have been previously described elsewhere.^20^, ^21^ Briefly, Kanagawa Prefecture, a metropolitan prefecture located next to Tokyo, is the second largest prefecture in Japan, and KCR is one of the largest local cancer registries in Japan. Well‐trained tumor registrars certified by the training program of the Japanese Association of Cancer Registries, whose program is accredited by SEER, are responsible for data collection. The data included basic information (sex, age, date of diagnosis, and date of death/last follow‐up) and clinical information (pathology, stage, and treatment). Uniquely, KCR collected information on occupation and smoking behaviors, if available, during the study period (approximately 10% of the registered cases); however, these data are no longer obtained since 2016 because of a change of data management practice. KCR automatically updates dates of death/last follow‐up with population registers and death certificates, and previous diagnosis codes, as well as pathological codes, are updated to be consistent with changes in coding practice. We obtained a deidentified data set under the research agreement between the authors and KCR, and the research ethics committee of The University of Tokyo, Tokyo (Protocol Number 3891‐4), approved the study.

We included all 7525 RCC patients registered in the KCR (a) who were diagnosed with RCC (C64 in International Classification of Diseases, 10th revision) between 2004 and 2014, (b) aged 20 and above, and (c) who had complete observation duration. We excluded those who had missing data for pathological type.

### 2.2 Histological subtype and 5‐year overall survival

Using pathology codes (identified by International Classification of Disease for Oncology, Third edition [ICD‐O‐3] pathology codes), we distinguished the histological subtypes of clear cell (ICD‐O‐3 codes of 8310, 8312, 8316, and 8320), papillary (ICD‐O‐3 code of 8260), and chromophobe (ICD‐O‐3 codes of 8270 and 8317) from “others” subtype (eg, collecting duct and sarcomatoid differentiation), according to previous SEER studies.^6^, ^8^, ^22^

The 5‐year overall survival was defined by the right‐censored, observation duration (person‐years) from the date of initial diagnosis to the date of death/last follow‐up (median follow‐up time, 2.1 years).

### 2.3 Covariates

The age (1‐year age category) and year of diagnosis (calendar year) were adjusted as continuous variables across all statistical models. To control for secular changes in clinicopathological diagnosis and treatment regimens, including surgery and systemic therapies, over time, we adjusted for year of diagnosis. Additionally, clinicopathological variables were included in survival analyses as potential mediating variables, ie, these variables do not confound the association between sex and RCC survival, but rather, they may help to explain the observed differences. Our clinicopathological variables of interest included the following^3^, ^4^, ^5^, ^6^, ^17^, ^20^, ^21^, ^22^: WHO pathological grade (grades 3 or 4 [high‐grade] vs grades 1 or 2 [low‐grade]), the Union for International Cancer Control TNM stage (stages III and IV [late stage] vs stages I and II [early stage]), and any performed surgeries including radical/partial nephrectomy (yes/no), as well as SES (high SES [with the longest‐held occupational class of managerial or professional workers] vs low SES) and smoking habits (never/ever).

### 2.4 Statistical analysis

The background characteristics between men and women were compared by *t* test or chi‐squared test. The 5‐year overall survival rates were estimated with Kaplan‐Meier curves and compared by log‐rank test. Except for basic characteristics (age, sex, year of diagnosis, and survival time) and histological subtypes, records included missing data on mediating factors: pathological grade (1897, 36.0%), stage (1474, 28.0%), surgery (343, 6.5%), SES (4906, 93.2%), and smoking habits (3662, 69.6%). Excluding patients with missing data may lead to biased inference; therefore, we conducted multiple imputation for missing data among the 5265 study subjects with all variables used for analysis, and 20 imputed data sets were generated by the Multiple Imputation by Chained Equations method (Table 1).^3^, ^4^, ^5^, ^23^

**Table 1:** Characteristics of renal cell carcinoma patients in Kanagawa Cancer Registry

Characteristics	Mean (SD) or Number (%)^a^						
	Crude				Multiple Imputation^c^		
	All n = 5265	Men n = 3820	Women n = 1445	*P* b	All n = 5265	Men n = 3820	Women n = 1445
Basic characteristics							
Histology^d^							
Clear cell	4642 (88.2%)	3383 (88.6%)	1259 (87.1%)	.15	88.2%	88.6%	87.1%
Papillary	217 (4.1%)	176 (4.6%)	41 (2.8%)	.004	4.1%	4.6%	2.8%
Chromophobe	129 (2.5%)	60 (1.6%)	69 (4.8%)	<.001	2.5%	1.6%	4.8%
Others	277 (5.3%)	201 (5.3%)	76 (5.3%)	>.99	5.3%	5.3%	5.3%
Age, y	64 (12)	64 (12)	65 (12)	<.001	64 (12)	64 (12)	65 (12)
Year of diagnosis	2009 (3)	2009 (3)	2009 (3)	.23	2009 (3)	2009 (3)	2009 (3)
Other backgrounds^e^							
Pathological grade	n = 3,368	n = 2,424	n = 944				
High grade	355 (10.5%)	257 (10.6%)	98 (10.4%)	.85	12.6%	12.5%	12.7%
Stage	n = 3791	n = 2753	n = 1038				
Late stage	983 (25.9%)	724 (26.3%)	259 (25.0%)	.40	32.9%	33.1%	32.3%
Treatment	n = 4922	n = 3562	n = 1360				
Surgery	4656 (94.6%)	3368 (94.6%)	1288 (94.7%)	.83	94.2%	94.1%	94.2%
SES	n = 359	n = 316	n = 43				
High‐SES	73 (20.3%)	62 (19.6%)	11 (25.6%)	.36	27.5%	27.7%	27.1%
Smoking	n = 1603	n = 1162	n = 441				
Ever smoker	789 (49.2%)	712 (61.3%)	77 (17.5%)	<.001	49.6%	61.8%	17.4%
5‐y overall survival, %							
Overall	72.0%	72.0%	71.9%	.95	72.0%	72.0%	71.9%
Clear cell	74.2%	74.0%	74.8%	.61	74.2%	74.0%	74.8%
Papillary	75.4%	76.7%	70.1%	.45	75.4%	76.7%	70.1%
Chromophobe	89.0%	91.8%	86.1%	.48	89.0%	91.8%	86.1%
Others	30.3%	33.3%	22.6%	.06	30.3%	33.3%	22.6%

Abbreviation: SES, socioeconomic status.

a Percentage may not total 100 because of rounding and multiple imputation.

b *P* values are for chi‐squared test or *t* test.

c Data were estimated with 20 imputed data sets. The number of missing data was, respectively, as follows: pathological grade (1897, 36.0%), stage (1474, 28.0%), surgery (343, 6.5%), SES (4906, 93.2%), and smoking habits (3662, 69.6%).

d The distribution of all histological subtypes combined differed between men and women (chi‐squared test, *P* < .001).

e Missing data are included.

In our main analytic model for the sex difference in histology (model 1), prevalence ratio (PR) and 95% confidence interval (CI) for each histological subtype were estimated by Poisson regression with robust variance, adjusted for age and year of diagnosis.^23^ Male patients served as the reference group for all analyses. In a Poisson regression with multiple imputation, we further controlled for pathological grade and stage (model 2) and SES and smoking habits (model 3) as potential mediating variables. In prior analyses, according to the methodology used in previous studies (multinomial logistic regression model),^3^, ^9^ we estimated multinomial odds ratios (in other word, relative risk ratios) for each type of RCC against clear cell subtypes among women compared with men. The magnitude and direction of odds ratios for each histological subtype were almost the same to the PRs for each histological subtype. However, we chose PRs for the final analytic method because the “prevalence” of each type of RCC among all primary RCC would be more intuitively rational compared with “odds” of each type of RCC against a specific RCC (ie, clear cell).

For sex differences in the RCC survival, HRs and 95% CIs for overall death were estimated by Cox proportional hazard model, adjusted for age and year of diagnosis (model 1). Male patients served as the reference group for all analyses. In a Cox regression with multiple imputation, we further controlled for histological subtypes (model 2) and pathological grade and stage (model 3) as potential mediating variables. Finally, in the maximally adjusted model, we controlled for all potential covariates (histological subtypes, pathological grade, stage, surgery, SES, and smoking habits, model 4).

In sensitivity analyses, because of the potential background differences between those who completed histological subtypes and those who did not complete histological subtypes, we performed regression analyses among all 7525 RCC patients (including 2260 patients who did not have complete histological information) with multiple imputation. Additionally, complete case analyses were performed. In the complete case analysis, SES was excluded from covariates because of the small sample size for the complete data. Alpha was set at .05, and all *P* values were two sided. Data were analyzed using STATA/MP13.1 (StataCorp LP, College Station, Texas).

## 3 RESULTS

From all 7525 RCC patients registered in the KCR who were aged 20 years and above (mean age [SD], 66 [13] y), we excluded those with missing data on pathological type (2260 patients, 30.0%), leaving a retrospective cohort comprising 5265 RCC patients (male 3820 [72.6%], female 1445 [27.4%]) for analysis. The percentage of missing data for pathology differed between men and women (male 28.8%, female 33.0%, *P* < 0.001, chi‐squared test). Among all 7525 RCC patients, the percentages of microscopic verification and Death Certificate Only were 70.0% and 11.9%, respectively.

For histological subtypes, the distribution differed between men and women (Table 1). The prevalence of clear cell and “others” subtype was similar between men and women (clear cell, male 88.6%, female 87.1%; “others,” male 5.3%, female 5.3%). However, the papillary subtype was less prevalent among women compared with men (male 4.6%, female 2.8%, *P* = .004, chi‐squared test), while chromophobe subtype was more prevalent among women compared with men (male 1.6%, female 4.8%, *P* < 0.001, chi‐squared test; Table 1). Except for histological subtypes, age, and smoking habits, background characteristics and 5‐year overall survivals did not show a statistically significant difference between men and women (Table 1).

In Poisson regression with robust variance, although the maximally adjusted PR of clear cell subtype showed a marginally lower prevalence in women (PR = 0.97, 95% CI, 0.96‐0.996, model 3), the PRs of clear cell subtype in model 1 and model 2 did not significantly differ between men and women (Table 2). Papillary subtype was less prevalent in women compared with men (model 1, Table 2): PR = 0.63 (95% CI, 0.45‐0.88). By contrast, chromophobe subtype was more prevalent in women (PR = 3.18; 95% CI, 2.26‐4.47). Even in the maximally adjusted model, papillary subtypes remained less prevalent, but chromophobe subtypes remained more prevalent in women compared with men (model 3, Table 2). The PR of the “others” subtype did not differ between men and women (Table 2).

**Table 2:** Prevalence ratios for each histological subtype estimated by Poisson regression with robust variance

Characteristics	Prevalence Ratio (95% Confidence Interval), n = 5265					
	Model 1	*P*	Model 2^a^	*P*	Model 3^a^	*P*
Clear cell						
Women	0.98 (0.96, 1.01)	.15	0.98 (0.96, 1.01)	.13	0.97 (0.94, 1.00)	.03
Age, continuous	1.00 (1.00, 1.00)	.63	1.00 (1.00, 1.00)	.78	1.00 (1.00, 1.00)	.92
Year of diagnosis, continuous	0.99 (0.99, 0.99)	<.001	0.99 (0.99, 0.99)	<.001	0.99 (0.99, 0.99)	<.001
High grade			0.74 (0.69, 0.80)	<.001	0.74 (0.69, 0.80)	<.001
Late stage			0.98 (0.94, 1.02)	.35	0.98 (0.95, 1.02)	.42
High SES					1.00 (0.94, 1.07)	.98
Ever smoker					0.97 (0.93, 1.01)	.11
Papillary						
Women	0.63 (0.45, 0.88)	.007	0.62 (0.44, 0.87)	.006	0.67 (0.46, 0.98)	.04
Age, continuous	1.00 (0.99, 1.01)	.90	1.00 (0.99, 1.01)	.97	1.00 (0.99, 1.01)	.98
Year of diagnosis, continuous	1.18 (1.12, 1.23)	<.001	1.16 (1.10, 1.22)	<.001	1.16 (1.10, 1.22)	<.001
High grade			2.04 (1.38, 3.03)	<.001	2.03 (1.36, 3.02)	<.001
Late stage			0.65 (0.45, 0.95)	.03	0.64 (0.44, 0.94)	.02
High SES					0.98 (0.63, 1.52)	.91
Ever smoker					1.19 (0.83, 1.72)	.34
Chromophobe						
Women	3.18 (2.26, 4.47)	<.001	3.14 (2.23, 4.42)	<.001	3.30 (2.23, 4.87)	<.001
Age, continuous	0.98 (0.97, 1.00)	.009	0.98 (0.97, 1.00)	.02	0.98 (0.97, 1.00)	.03
Year of diagnosis, continuous	1.18 (1.11, 1.27)	<.001	1.16 (1.08, 1.25)	<.001	1.16 (1.07, 1.24)	<.001
High grade			1.35 (0.71, 2.55)	.36	1.36 (0.73, 2.55)	.34
Late stage			0.39 (0.21, 0.74)	.004	0.39 (0.21, 0.72)	.003
High SES					1.03 (0.48, 2.23)	.93
Ever smoker					1.11 (0.69, 1.81)	.66
Others						
Women	0.97 (0.75, 1.26)	.84	0.99 (0.78, 1.27)	.96	1.18 (0.83, 1.67)	.35
Age, continuous	1.02 (1.00, 1.03)	.01	1.01 (1.00, 1.02)	.20	1.01 (1.00, 1.02)	.16
Year of diagnosis, continuous	0.99 (0.95, 1.03)	.59	1.00 (0.96, 1.04)	.96	1.00 (0.95, 1.04)	.94
High grade			6.21 (4.04, 9.53)	<.001	6.21 (4.06, 9.50)	<.001
Late stage			2.55 (1.38, 4.73)	.004	2.47 (1.35, 4.51)	.004
High SES					0.95 (0.43, 2.13)	.90
Ever smoker					1.47 (0.86, 2.51)	.15

Abbreviation: SES, socioeconomic status.

a Data were estimated with 20 imputed data sets.

In survival analyses, although the 5‐year overall survival rate was 72% in this population, patients with the “others” subtype had a poor prognosis (30.3%, Table 1). However, no statistically significant differences were observed between men and women, even after stratifying by histological subtypes (Table 1). In the Cox regression analysis, although the chromophobe subtype predicted a better prognosis and the “others” subtype predicted a poor prognosis, only the “others” subtype predicted prognosis in the maximally adjusted model (HR 1.74; 95% CI 1.32‐2.30, model 4, Table 3). However, we did not observe a statistically significant difference in the 5‐year overall survival between men and women in model 1 through model 4 (Table 3).

**Table 3:** Hazard ratios for 5‐year overall survival estimated by Cox proportional hazard model

Characteristics	Hazard Ratio (95% Confidence Interval), n = 5265							
	Model 1	*P*	Model 2	*P*	Model 3^1^	*P*	Model 4^1^	*P*
Women	0.95 (0.82, 1.08)	.42	0.99 (0.86, 1.13)	.88	0.98 (0.84, 1.15)	.84	1.13 (0.94, 1.37)	.20
Age	1.04 (1.03, 1.04)	<.001	1.03 (1.03, 1.04)	<.001	1.03 (1.02, 1.04)	<.001	1.03 (1.02, 1.03)	<.001
Year of diagnosis	1.07 (1.04, 1.10)	<.001	1.08 (1.05, 1.10)	<.001	1.11 (1.07, 1.16)	<.001	1.13 (1.08, 1.17)	<.001
Histological subtypes								
Clear cell			1.00		1.00		1.00	
Papillary			0.84 (0.59, 1.19)	.32	0.84 (0.59, 1.20)	.34	0.89 (0.62, 1.28)	.53
Chromophobe			0.38 (0.19, 0.76)	.006	0.47 (0.23, 0.98)	.04	0.51 (0.25, 1.06)	.07
Others			4.69 (3.97, 5.53)	<.001	2.15 (1.64, 2.83)	<.001	1.74 (1.32, 2.30)	<.001
High grade					2.52 (2.03, 3.13)	<.001	2.43 (1.95, 3.03)	<.001
Late stage					4.30 (3.33, 5.54)	<.001	3.62 (2.77, 4.74)	<.001
Any surgery							0.27 (0.21, 0.35)	<.001
High SES							1.16 (0.74, 1.83)	.51
Ever smoker							1.26 (0.94, 1.70)	.12

Abbreviation: SES, socioeconomic status.

1 Data were estimated with 20 imputed data sets.

In sensitivity analyses, the observed patterns were almost identical to the main results (Table 4). The multinomial odds ratios of women for the papillary, chromophobe, and “others” subtypes were, respectively, 0.64 (95% CI, 0.45‐0.91), 3.25 (95% CI, 2.28‐4.64), and 0.99 (95% CI, 0.75‐1.30).

**Table 4:** Sensitivity analyses for sex differences in renal cell carcinoma histology and survival by Poisson regression with robust variance and Cox proportional hazard model

Prevalence Ratio (95% Confidence Interval)	All Patients (n = 7525)^a^	*P*	Complete Case (n = 790)^b^	*P*
Clear cell				
Women	0.97 (0.94, 1.01)	.12	0.97 (0.92, 1.02)	.29
Papillary				
Women	0.67 (0.46, 0.99)	.04	0.74 (0.31, 1.74)	.48
Chromophobe				
Women	3.12 (2.01, 4.87)	<.001	3.46 (1.49, 8.00)	.004
Others				
Women	1.07 (0.84, 1.37)	.57	1.19 (0.33, 4.35)	.79

Hazard ratio (95% confidence interval)	All patients (n = 7525)^c^	*P*	Complete case (n = 787)^d^	*P*
**Women**	1.11 (0.99, 1.24)	.08	1.30 (0.74, 2.25)	.36

a Data were estimated with 20 imputed data sets. The numbers of missing data were, respectively, as follows: histological subtypes (2260, 30%), pathological grade (4156, 55%), stage (3296, 44%), surgery (1481, 20%), socioeconomic status (7113, 94%), and smoking habits (5569, 74%). Adjusted for age, year of diagnosis, pathological grade, stage, socioeconomic status, and smoking habits.

b Adjusted for age, year of diagnosis, pathological grade, stage, and smoking habits.

c Data were estimated with 20 imputed data sets. Adjusted for age, year of diagnosis, histological subtype, pathological grade, stage, treatment, socioeconomic status, and smoking habits.

d Adjusted for age, year of diagnosis, histological subtype, pathological grade, stage, treatment, and smoking habits.

## 4 DISCUSSION

As far as we are aware, this is the first analysis of sex differences in histology and survival in RCC patients in Japan. Compared with men, although women did not have a significantly different prevalence for clear cell and “others” subtypes, women had different prevalence for papillary and chromophobe subtypes (0.6 times lower prevalence for papillary subtype and 3.2 times higher prevalence for chromophobe subtype). Even after controlling for potential mediating factors, the sex difference for these histological subtypes persisted. However, the survival was similar between men and women, even after accounting for survival differences by histological subtypes (eg, RCC patients with “others” subtype had 1.7 times poorer survival), as well as other potential prognostic factors.

Differences in female lifestyle and behavioral risks (obesity and smoking) between Western countries and Japan may underlie our observed sex difference in RCC histology of the clear cell subtype,^15^, ^17^ which differs from the pattern seen in Western countries.^8^, ^13^, ^22^ Although the prevalence of the clear cell subtype varies across populations and regions, studies in the United States and Europe consistently suggest a higher prevalence of the clear cell subtype among women, by approximately 2% to 7%.^8^, ^11^, ^12^, ^13^, ^14^, ^22^ However, the prevalence of the clear cell subtype was not higher among women compared with men in our study population in Japan, which partly coincides with the result from South Korea.^19^ Studies imply potential pathways of group‐based differences in the risk for the clear cell subtype via obesity, smoking, hypertension, and end‐stage renal disease in combination with genetic factors (APOL1 gene).^1^, ^12^, ^13^, ^15^, ^17^, ^24^ Since obesity and smoking habits are far less common among Asian women compared with their counterparts in Western countries,^16^, ^25^ the flat “gradient” of sex difference in the clear cell subtype seems plausible as an explanation for the discrepancy between the results obtained from Western and Asian countries.

The sex difference in the other RCC histological subtypes may be consistent with the data from Western countries. In the Western settings, previous studies suggested that the papillary subtype was less prevalent (OR approximately 0.5 to 0.6) but that the chromophobe subtype was more prevalent (OR 2.3) among women compared with men.^12^, ^13^ In our population‐based study in Japan, we confirmed this pattern with similar magnitudes and directions (PRs for papillary and chromophobe subtypes were, respectively, 0.63 and 3.18). Some biological mechanisms, eg, androgen receptor expression,^26^ may play a role. Yet, this sex difference is not well‐characterized via biological pathways. In addition, the prevalence of the papillary (4.1%) and chromophobe (2.5%) subtypes in the total analyzed population, which parallels the statistics of the Japanese Urological Association,^18^ is likely at the lower end of published estimates compared with data from previous studies,^6^, ^7^, ^8^, ^12^, ^13^, ^19^ suggesting regional disparity in RCC histology.

Regarding sex disparities in RCC survival, women have been previously reported to have better prognosis compared with men,^11^, ^14^, ^19^ which we did not observe in the present study. In the Western settings, better prognostic factors, including smaller tumor size, low pathological grade, and early stage, are likely more prevalent among women compared with men.^11^, ^14^, ^27^ However, in this non‐Western setting, we did not observe a sex difference in grade/stage. Similarly, a sex difference for grade/stage/tumor size was not found in South Korea.^19^ The similar distributions of better prognostic factors between men and women might partly underlie the observed absence of sex disparity in RCC survival in our study, as opposed to studies in the Western setting.^11^

Several limitations in this study should be noted. First, although our data set was population‐based, it only represents approximately 7% of the Japanese population in one geographic region, and our obtained pathology diagnoses were not based on a central pathology review. In addition, other relevant outcomes (eg, relative survival)^28^ were not evaluated, and complete data were limited for histology and other prognostic variables (including stage) because of missing data, thereby limiting internal and external generalizability. However, our sensitivity analyses with multiple imputation and complete data yielded almost identical results. Second, although we assessed SES and smoking habits, we could not assess other potential predicting factors such as metabolic disorders (eg, obesity, hypertension, and diabetes), tumor size, or performance status, as seen in previous studies.^1^, ^6^, ^10^, ^11^, ^19^ However, hypertension and diabetes did not affect prognosis in a previous study in South Korea.^19^ Third, because of the limitation of the data, we were not able to classify histological subtypes further (such as type 1 and type 2 papillary RCC).^28^ Since patients with type 2 papillary RCC tend to have poorer prognosis,^29^ future studies focusing on sex differences in papillary RCC subtypes are needed.

Despite these limitations, the strengths of our study included the size, as this is one of the most extensive studies conducted for evaluating sex differences in RCC histology in the non‐Western setting. Our distribution of the clear cell subtype (88.2%) estimated with population‐based data was similar to the SEER data,^6^, ^7^, ^11^, ^21^ suggesting our reduced bias compared with nonpopulation‐based studies.^18^, ^19^ In addition, while previous population‐based studies did not include SES or smoking habits,^11^ we were able to take account of these characteristics in our study.

Lastly, RCC patients with the “others” subtype tend to have a poorer prognosis compared with those with the clear cell subtype,^8^, ^30^ and we confirmed this disparity in Japan. In contrast to good prognoses in clear cell, papillary, and chromophobe subtypes (even though survivals tend to differ slightly among the three subtypes),^7^ prognosis of the “others” subtype remains poor, with aggressive pathological features (eg, approximately 50% of RCC patients having the “others” subtype tend to present with metastasis).^8^ Hence, further studies exploring effects of standard and novel agents for this high‐risk population are warranted.^29^

In conclusion, sex differences in RCC histology (papillary and chromophobe subtypes but not clear cell and other subtypes) appear to exist in Japan, which differs from the pattern previously described in Western countries. Sex differences in RCC histology may not affect RCC survival in this population. Further understanding of RCC etiology from an integrated perspective of social and clinicopathological epidemiology may elucidate the determinants of sex differences in RCC histology and prognosis.

## FUNDING

This work received funding from the Ministry of Health, Labour and Welfare (Industrial Disease Clinical Research Grants 170201‐01) and Japan Society for the Promotion of Science (JSPS KAKENHI JP18K17351). The supporting source had no involvement in study design; collection, analysis, and interpretation of data; writing of the report; and the decision to submit the report for publication.

## CONFLICT OF INTEREST

The authors declare no potential conflicts of interest.

## AUTHOR CONTRIBUTIONS

Conceptualization: Masayoshi Zaitsu, Ichiro Kawachi

Formal Analysis: Masayoshi Zaitsu

Funding acquisition: Masayoshi Zaitsu, Yasuki Kobayashi

Methodology: Masayoshi Zaitsu, Satoshi Toyokawa

Study Supervision: Masayoshi Zaitsu, Yasuki Kobayashi, Ichiro Kawachi

Writing – Original Draft Preparation: Masayoshi Zaitsu

Writing – Review & Editing: Masayoshi Zaitsu, Satoshi Toyokawa, Takumi Takeuchi, Yasuki Kobayashi, Ichiro Kawachi

All authors have read and approved the final version of the manuscript. Masayoshi Zaitsu had full access to all of the data in this study and takes complete responsibility for the integrity of the data and the accuracy of the data analysis.

## TRANSPARENCY STATEMENT

Masayoshi Zaitsu affirms that this manuscript is an honest, accurate, and transparent account of the study being reported, that no important aspects of the study have been omitted, and that any discrepancies from the study as planned (and, if relevant, registered) have been explained.

## Data Availability

The data that support the findings of this study are available from the Kanagawa Cancer Registry. Restrictions apply to the availability of these data, which were used under license for this study by the Kanagawa Cancer Registry; research data used in the study cannot be made publicly available directly by the authors. If any person wishes to verify our data analysis, they are most welcome to contact the corresponding author.
